# Identification of CBF Transcription Factors in Tea Plants and a Survey of Potential CBF Target Genes under Low Temperature

**DOI:** 10.3390/ijms20205137

**Published:** 2019-10-17

**Authors:** Pengjie Wang, Xuejin Chen, Yongchun Guo, Yucheng Zheng, Chuan Yue, Jiangfan Yang, Naixing Ye

**Affiliations:** Key Laboratory of Tea Science, College of Horticulture, Fujian Agriculture and Forestry University, Fuzhou 350002, China; 2180311002@fafu.edu.cn (P.W.); 1180311002@fafu.edu.cn (X.C.); 1180311006@fafu.edu.cn (Y.G.); 1170311017@fafu.edu.cn (Y.Z.); yuec@fafu.edu.cn (C.Y.)

**Keywords:** C-repeat binding factors, *Camellia sinensis*, low temperature, target genes

## Abstract

C-repeat binding factors (CBFs) are key signaling genes that can be rapidly induced by cold and bind to the C-repeat/dehydration-responsive motif (CRT/DRE) in the promoter region of the downstream cold-responsive (COR) genes, which play a vital role in the plant response to low temperature. However, the CBF family in tea plants has not yet been elucidated, and the possible target genes regulated by this family under low temperature are still unclear. In this study, we identified five *CsCBF* family genes in the tea plant genome and analyzed their phylogenetic tree, conserved domains and motifs, and cis-elements. These results indicate that *CsCBF3* may be unique in the *CsCBF* family. This is further supported by our findings from the low-temperature treatment: all the *CsCBF* genes except *CsCBF3* were significantly induced after treatment at 4 °C. The expression profiles of eight tea plant tissues showed that *CsCBFs* were mainly expressed in winter mature leaves, roots and fruits. Furthermore, 685 potential target genes were identified by transcriptome data and CRT/DRE element information. These target genes play a functional role under the low temperatures of winter through multiple pathways, including carbohydrate metabolism, lipid metabolism, cell wall modification, circadian rhythm, calcium signaling, transcriptional cascade, and hormone signaling pathways. Our findings will further the understanding of the stress regulatory network of *CsCBFs* in tea plants.

## 1. Introduction

Low temperature is a major abiotic stress that limits the distribution, growing season, and production of plants worldwide. Plants in temperate climatic regions need to undergo a period of rapid temperature change between warm and cold seasons. When plants encounter nonfreezing low temperatures, their chilling and freezing tolerance can be increased, which is an adaptive process known as cold acclimation [1,2,3]. Many plants, such as Arabidopsis, canola, and wheat, have evolved a series of complex cold acclimation mechanisms that involve a wide range of physiological, biochemical and metabolic changes [4,5,6,7]. These include the accumulation of carbohydrate and cryoprotective compounds, changes in lipid compositions, and modifications in membranes, cell walls, and cytoskeleton stability [2,8,9]. Accumulating evidence demonstrates that most of these changes in low-temperature conditions are the result of multiple cold-responsive (COR) gene expression [10,11,12].

In recent years, the molecular mechanism of cold acclimation has been extensively studied and is dedicated to finding key regulatory factors for this complex network. Among them, the C-repeat binding factors (CBFs) can be rapidly induced by cold and bind to the C-repeat/dehydration-responsive motif (CRT/DRE; G/ACCGAC) in the promoter region of downstream COR genes, which play vital roles in the response of plants to low temperature [13,14,15]. CBFs belong to a small family of APETALA2/Ethylene-Responsive Factor (AP2/ERF) transcription factor superfamily with a highly conserved AP2 DNA-binding domain and two signature sequences (PKK/RPAGRxKFxETRHP and DSAWR) [16,17]. There are four CBF members in *Arabidopsis*, of which *AtCBF1* (*DREB1B*), *AtCBF2* (*DREB1C*), and *AtCBF3* (*DREB1A*) have been shown to be widely involved in the regulation of plant cold stress [5,13,15,18], while *AtCBF4* (*DREB1D*) is differentiated from the other members and plays a role in plant drought stress tolerance [19]. In addition, the CBF genes have been isolated in many other species, such as rice [20], wheat [21], barley [22], grape [23], tomato [24], poplar [25], and tea plant [26,27], and their functional roles in regulating cold tolerance across diverse plant species have also been characterized.

Previous reports have identified many potential CBF target genes [5,18,28,29,30]. Gene arrays have indicated that the overexpression of *AtCBF1*, *AtCBF2* and *AtCBF3* in *Arabidopsis* activates a set of 30 identical target genes [29]. Transcriptome analysis of AtCBF-overexpressing lines under low temperature treatment revealed that 133 COR genes are activated by CBFs, and 39 are repressed [18]. Moreover, 346 CBF-activated COR genes were discovered by the cbf triple CRISPR mutants, and 212 (61.3%) were found to contain CRT/DRE motifs, and their functional annotations showed that most of the COR genes involved carbohydrate and lipid metabolism, cell wall organization or biogenesis, and hormone signal transduction [5]. A recent study of perennial woody poplars found 2263 potential CBF target genes through genome-wide searches that are involved in a variety of biological processes and pathways [30].

Tea plant, *Camellia sinensis* (L.) O. Kuntze, an important perennial woody plant, is widely distributed in subtropical to tropical climate regions [31]. Natural winter low temperature is one of the most essential environmental factors restricting the geographical position, growth and yield of tea plants [26]. Previous studies have shown that carbohydrate metabolism and calcium signaling play vital roles during cold acclimation [31,32], and cell wall modification is found to improve freezing tolerance in tea plants [33]. Comprehensive transcriptional and metabolic analyses indicated that MAPK-dependent ethylene and calcium signaling pathways and the ICE-CBF-COR cascade were involved in the spring cold spell response in young shoots of the tea plant [34]. Recently, with the publication of high-quality genome sequencing data for tea plants (vars. *assamica* and *sinensis*) [35,36], a growing number of transcription factor families involved in cold tolerance have been identified [37,38,39,40,41]. However, the critical CBF transcription factor family in tea plants has not yet been revealed, and the possible target genes regulated by this family under low temperature stress remain to be elucidated. In this study, we identified the *CsCBF* gene family in tea plants by exploring the latest genomic data. A phylogenetic analysis of the CsCBF protein sequences from tea plants and Arabidopsis was performed, and their conserved domains and motifs were compared. Furthermore, we analyzed the promoter, tissue-specific and low-temperature response expression patterns of *CsCBFs* in tea plants and identified, annotated and classified the putative target genes of *CsCBFs* under natural low-temperature conditions.

## 2. Results

### 2.1. Identification and Characterization of CsCBFs

We obtained five CsCBF genes in tea plants and named them according to their order in the genome (Table 1). Among the five genes, CsCBF5 is identical to the previously reported sequence [26], and the remaining four are newly identified CsCBF genes. The sequence characteristics analysis revealed that the coding sequences (CDS) of CsCBFs ranged from 540 bp to 1056 bp, with deduced proteins of 179 to 351 amino acids. The molecular weight (MW) of the CsCBF proteins varied from 19.54 kDa to 38.28 kDa, with an isoelectric point (pI) ranging from 4.75 to 9.54. The grand average of hydropathicity (GRAVY) value of CsCBF proteins varied from −0.59 to −0.28, all less than 0, indicating that they are hydrophilic proteins. Furthermore, subcellular localization predictions indicate that CsCBF family proteins are localized in the nucleus. More detailed information on CsCBFs, including their nucleotide and amino acid sequences, instability indices and aliphatic indices, is listed in Table 1 and Table S1.

The instability index provides an estimate of the stability of the protein in a test tube, A protein whose instability index is smaller than 40 is predicted as stable, a value above 40 predicts that the protein may be unstable. The aliphatic index of a protein is defined as the relative volume occupied by aliphatic side chains (alanine, valine, isoleucine, and leucine). The GRAVY value for a protein is calculated as the sum of hydropathy values of all the amino acids, divided by the number of residues in the sequence.

### 2.2. Comparative Analysis of the DNA-Binding Domain, Phylogenetic Tree and Conserved Motifs

**Figure 1:**
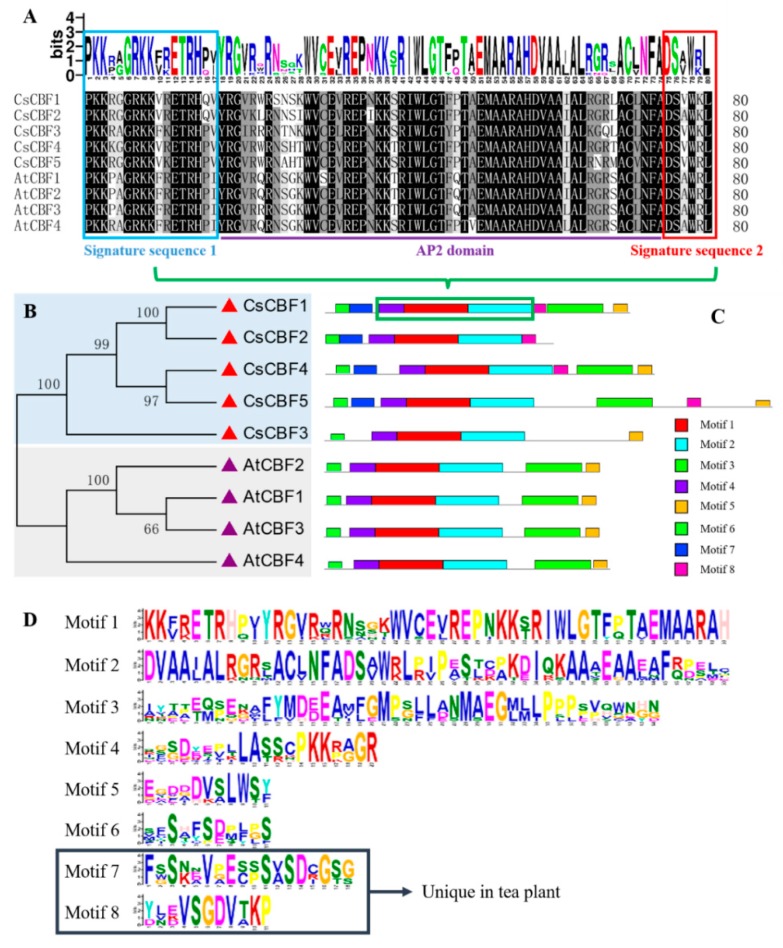
Sequence alignment, phylogenetic and conserved motif analysis of CsCBFs and AtCBFs. (**A**) Multiple sequence alignment of the AP2 DNA-binding domain and two signature sequences and generating a logo. The *Y* axis (measured in bits) of logo depicts the overall height of the stack, indicating the sequence conservation at that position, while the height of symbols within the stack indicates the relative frequency of each amino at that position. The purple underline indicates the AP2 DNA-binding domain; the blue box represents the PKK/RPAGRxKFxETRHP signature sequence, and the red box represents the DSAWR signature sequence. (**B**) Phylogenetic analysis of the CBF protein sequences from tea plants and Arabidopsis. The red triangle represents the tea plants and the purple triangle represents the Arabidopsis. The phylogenetic tree was constructed using MEGA 5.0 software and the Neighbor-Joining method. The number above/below the branch represents the bootstrap values (from 1000 replicates). (**C**) Eight conserved motifs were predicted by MEME. (**D**) Detailed information of the eight conserved motifs.

To examine the DNA-binding domain characteristics of CsCBF proteins, multiple sequence alignments were performed between tea plants and Arabidopsis (Figure 1A). The domain sequences of CsCBFs and AtCBFs were highly conserved (with 89% sequence identity), including the AP2 DNA-binding domain and two flanking signature sequences (PKK/RPAGRxKFxETRHP and DSAWR). Slight variations were found in some amino acids, which were conserved separately in tea plants and Arabidopsis. We further constructed a phylogenetic tree of CBF proteins from tea plants and Arabidopsis (Figure 1B). The results showed that the CBF proteins were distinctly classified into two clades of CsCBFs and AtCBFs, suggesting that the CBFs were phylogenetically conserved across these two plant species. Furthermore, CsCBF3 and AtCBF4 in different clades were distinguished from other homologous genes by clustering into different subclades, indicating that they might be functionally differentiated. We further analyzed the conserved motifs of CsCBFs and AtCBFs, and eight motifs were presented and named as motif 1 to motif 8 (Figure 1C,D). Motif 1, motif 2 and motif 4 contain the AP2 DNA binding domain and two flanking signature sequences and were present in all CBFs. It is notable that motif 7 and motif 8 were found exclusively in CsCBFs, indicating that CsCBFs were evolutionarily differentiated from AtCBFs.

### 2.3. Bioinformatics Analysis of Cis-Elements

**Figure 2:**
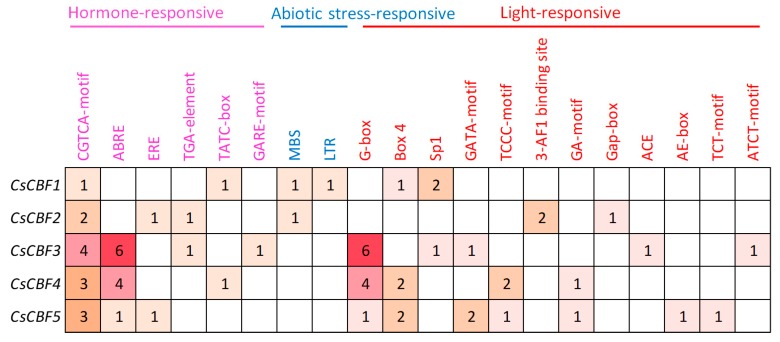
Prediction of cis-elements in the CsCBF gene promoters. The numbers of different cis-elements in the CsCBFs are indicated by numbers and different colors in the grid.

To investigate the functional roles of the cis-elements of the CsCBF promoters, the 1000 bp upstream sequences of CsCBFs were analyzed. A total of 61 types of cis-elements were identified in the CsCBF promoters (Table S2), including 6 hormone-responsive, 2 abiotic stress-related, and 12 light-sensitive elements (Figure 2). The light-sensitive cis-elements comprised the largest part of all elements, including the G-box, Box 4, Sp1, GATA-motif, TCCC-motif, 3-AF1 binding site, GA-motif, Gap-box, ACE, AE-box, TCT-motif, and ATCT-motif. The CGTCA motif in response to MeJA and the ABRE in response to ABA were the most abundant hormone-responsive elements in CsCBF promoters. Furthermore, a small number of hormone-responsive cis-elements, such as ERE (ethylene), TGA-element (auxin), TATC-box and GARE-motif (gibberellin), and abiotic stress-responsive cis-elements, such as MBS (drought) and low temperature (LTR), were detected.

### 2.4. Expression Profiles of CsCBFs in Eight Tea Plant Tissues

**Figure 3:**
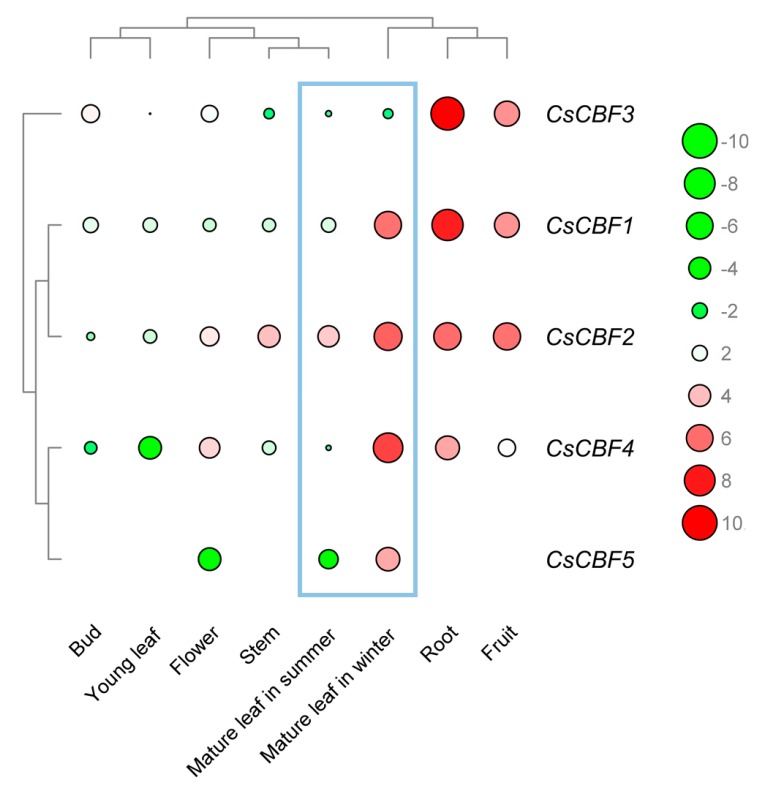
Expression of CsCBF genes in eight tea plant tissues. The bar color represents the normalized FPKM values, green for low expression, red for high expression, and no expression for null. Circle sizes represent the levels of expression. The blue box represents that the expression level of the CsCBF genes is substantially different between mature leaves in winter and mature leaves in summer.

To examine the potential role of CsCBFs in the tea plant tissues, the RNA-Seq data from eight tissues published along with the tea plant genome data were downloaded and analyzed (Table S3, Figure 3). The expression level of CsCBF genes showed significant tissue specificity. For example, CsCBF1, CsCBF2, CsCBF4, and CsCBF5 were highly expressed in mature leaves in winter (FPKM > 10), and the transcripts of CsCBF1, CsCBF2, and CsCBF3 significantly accumulated in the roots and fruits (FPKM > 10). Compared with those in mature leaves in summer, the expression levels of CsCBF1, CsCBF2, CsCBF4, and CsCBF5 increased significantly in mature leaves in winter, implying that the CsCBF genes play roles in low temperatures during the winter. In addition, the results of hierarchical clustering analysis were consistent with previous phylogenetic analysis, and the expression levels of CsCBF3 and other CsCBFs were clustered into two groups.

### 2.5. Expression Profiles of Cscbfs in Response to Low Temperature

**Figure 4:**
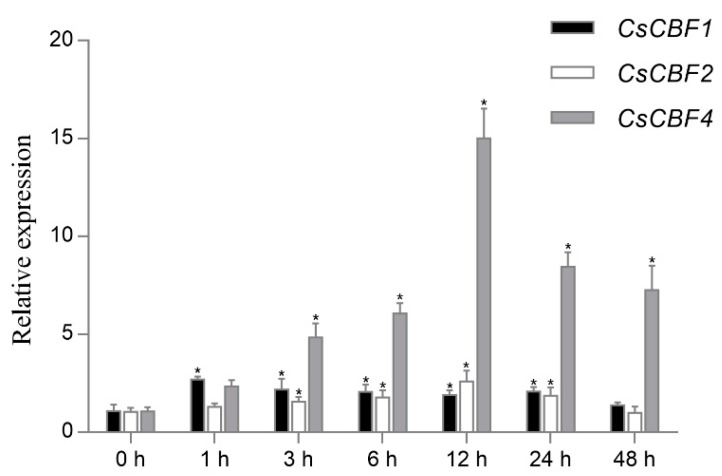
Expression of *CsCBF* genes under 4 °C treatment. The transcripts were analyzed by qRT-PCR. Error bars indicate the standard error of the mean (*n* = 3). Asterisks indicate statistically significant differences (*p* < 0.05). CsGAPDH was used as an internal control. The expression value is listed in Table S4.

To explore the role of the CsCBF genes in response to low temperature, we analyzed the expression profiles of the CsCBF genes under 4 °C treatment. However, since the expression level of CsCBF3 is too low to be reliably detected by qRT-PCR technology (also almost no accumulation in leaf tissues in the previously analyzed transcriptome data), and CsCBF5 was reported to accumulate immediately and reached a maximum after 6 h at 4 °C [26], only CsCBF1, CsCBF2, and CsCBF4 were analyzed further. As shown in Figure 4, the expression levels of three CsCBF genes were significantly upregulated after treatment at 4 °C. Among them, CsCBF1 showed peak induction at 1 h, while CsCBF2 and CsCBF4 reached their highest level at 12 h. These results suggest that the CsCBFs, except for CsCBF3, may play a critical role in the response to low temperature in tea plants.

### 2.6. Identification and Annotationo of Potential Cscbf Target Genes at Low Temperature in the Winter

To further identify the possible target genes regulated by CsCBFs under low-temperature conditions, two criteria were used: (1) The genes were upregulated or downregulated at least 2-fold in winter mature leaves compared to in summer mature leaves. (2) The promoter region of the genes has at least one CRT/DRE motif. A total of 685 potential target genes were identified, including 176 upregulated genes and 509 downregulated genes. These 685 target genes with at least one CRT/DRE motif in their putative promoters were used for further annotation. Of the 685 genes, 666 were annotated in the Nr database based on protein sequence homologies; 316 were annotated in the GO database, and 229 were mapped to the reference pathway in the KEGG database.

**Figure 5:**
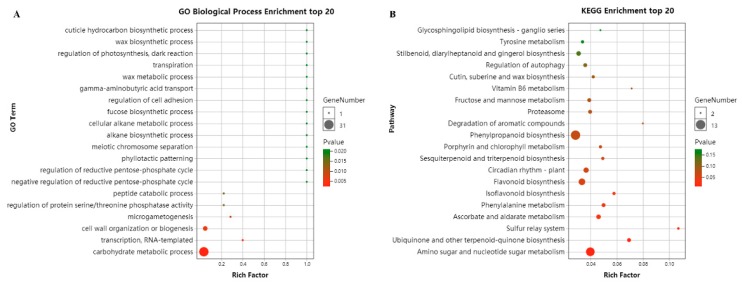
The top 20 GO terms (**A**) and KEGG pathways (**B**) for CsCBF target gene enrichment. The black circles indicate the number of target genes, and different colors indicate the level of *p* value, ranging from 0 to 1.

For the GO classification, the target genes were assigned into three major categories and 41 subcategories (Figure S1). GO analysis showed that many target genes were implicated in the terms “response to stimulus (GO:0050896)”, “response to stress (GO:0006950)”, and “response to hormone (GO:0009725)”. In the top 20 GO biological process enrichment analyses, typically the terms “carbohydrate metabolic process (GO:0005975)”, “transcription, RNA-templated (GO:0001172)”, and “cell wall organization or biogenesis (GO:0071554)” were significantly enriched (Figure 5A).

For the KEGG analysis, most of the target genes were enriched in the metabolism pathway (Figure S2). The top 20 KEGG pathway enrichment analyses showed that the pathways “Amino sugar and nucleotide sugar metabolism (ko00520)”, “Ubiquinone and other terpenoid-quinone biosynthesis (ko00130)”, “Sulfur relay system (ko04122)”, “Ascorbate and aldarate metabolism (ko00053)”, “Phenylalanine metabolism (ko00360)”, and “Isoflavonoid biosynthesis (ko00943)” were significantly enriched (*p* < 0.05) (Figure 5B). Furthermore, the target genes were also enriched in the pathways “Flavonoid biosynthesis (ko00941)”, “Circadian rhythm – plant (ko04712)”, “Sesquiterpenoid and triterpenoid biosynthesis (ko00909)”, “Porphyrin and chlorophyll metabolism (ko00860)”, “Phenylpropanoid biosynthesis (ko00940)”, “Degradation of aromatic compounds (ko01220)”, and “Cutin, suberin and wax biosynthesis (ko00073)”. These results indicate that the target genes regulated by CsCBFs play a functional role through multiple pathways in winter low temperature.

### 2.7. Functional Analysis of the CsCBF Putative Target Genes

**Figure 6:**
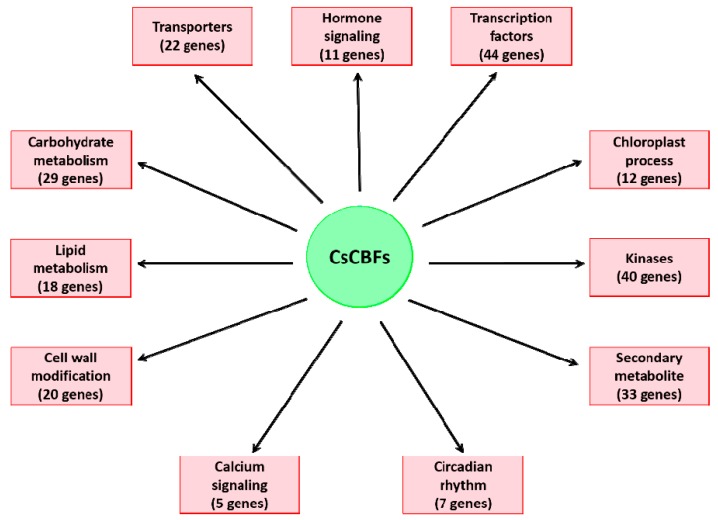
Functional categories of putative CsCBF target genes. The target genes were divided into 11 categories according to their biological functions. Their expression values and annotations are listed in Table S5.

**Figure 7:**
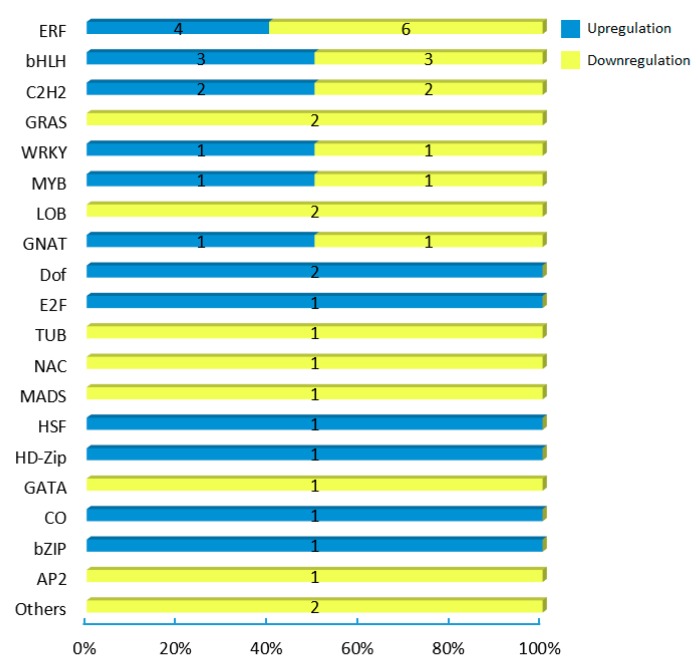
The distribution of putative target transcription factors of CsCBFs. Blue and yellow indicate significant up- and downregulation of the target genes, respectively. The numbers on bars represent the number of transcription factors, and the percentages on the horizontal axis represent the percentage of the number of transcription factors.

**Figure 8:**
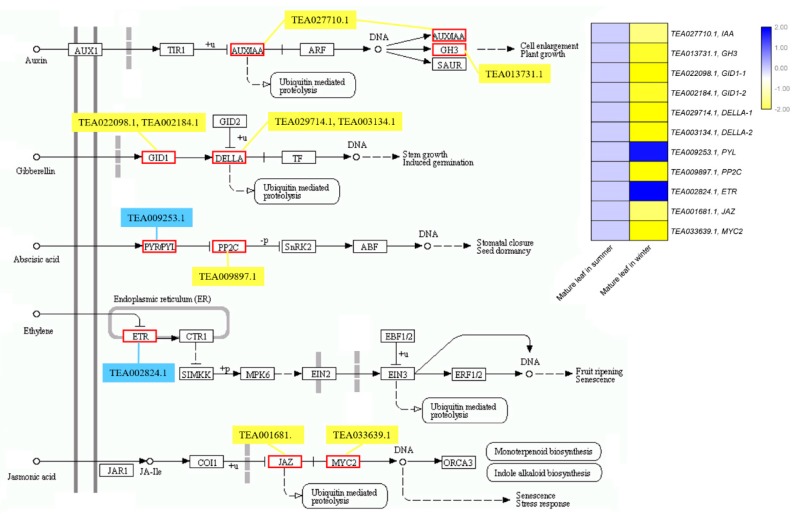
The putative target genes of CsCBFs involved in the plant hormone signaling pathways. The bar color represents the normalized FPKM values. Blue and yellow indicate significant up- and downregulation of target genes, respectively.

To explore the regulatory network of the putative CsCBF target genes under the low temperatures of winter, we further analyzed the target genes and divided them into 11 functional categories (Figure 6 and Table S5). We identified 29 target genes related to carbohydrate metabolism, including raffinose synthases, β-glucosidases, β-galactosidases, and galactinol synthases. The cold-induced expression of the galactinol synthase-encoding gene GOLS3 has been reported to be completely dependent on AtCBFs. Here, three GOLS genes (TEA006791.1, TEA006804.1, and TEA031908.1) were found to increase by approximately 4- to 39-fold in winter mature leaves. Furthermore, we found 18 lipid metabolism-related genes, including wax-ester synthase (TEA021142.1), phospholipases (TEA026635.1 and TEA031004.1), and aldehyde decarboxylase (TEA032831.1). Another large group of target genes of CsCBFs were involved in cell wall modification, including genes encoding chitinases (TEA024621.1, TEA024667.1, and TEA026337.1), pectate lyase (TEA010416.1), cellulose (TEA020572.1), polygalacturonases (TEA021750.1, TEA024110.1, and TEA024124.1), and xyloglucans (TEA008094.1, TEA008214.1, and TEA020211.1). The calcium signaling pathway might play a crucial role during cold acclimation in tea plants [31]. We found that five target genes are associated with the calcium signaling pathway: these genes encode two Ca2+-transporting ATPases (TEA025095.1, TEA027208.1), two pore calcium channel proteins (TEA018313.1), and annexin (TEA032977.1). A total of 44 putative target transcription factors were regulated by CsCBFs, of which 19 were significantly upregulated, and 25 were downregulated (Figure 7). These transcription factors include multiple members of the ERF, bHLH, C2H2, GRAS, WRKY, MYB, LOB, GNAT, and Dof transcription factor families, suggesting that some transcriptional cascades may participate in the cold regulatory network. Eleven CsCBF target genes were identified that are involved in the plant hormone signaling pathways of auxin, gibberellin (GA), abscisic acid (ABA), ethylene (ETH), and jasmonic acid (JA), including 2 upregulated and 9 downregulated genes (Figure 8). Among these, six target genes were designated transcription factors (IAA, GH3, DELLA, JAZ, and MYC2) involved in the auxin, GA, and JA signaling pathways, respectively. These results indicate that cold stress is associated with plant hormonal responses. In addition, multiple target genes regulated by the CsCBF proteins were also involved in circadian rhythms, kinases, chloroplast processes, and transporters (Figure 6).

## 3. Discussion

Plants are often challenged by a variety of environmental stresses that restrict their growth and development, in which low temperature is one of the major constraints. Accumulated evidence indicates that the CBF pathway is the key signaling pathway involved in regulating the cold tolerance of plants [15,42]. It has been reported that CBFs exist in multiple copies in plants and exhibit a considerable degree of functional redundancy [5]. However, the CBF transcription factor family in tea plants has not been revealed, and its biological functions, especially the regulated target genes under low temperature, are still unclear. Here, we obtained five *CBF* genes in the tea plant genome, of which *CsCBF5* is identical to the previously published sequence [26], and the remaining four are newly identified *CsCBF* genes. The number of CBFs in tea plants is slightly more than that in *Arabidopsis* [15], but much less than that in many monocots [43,44,45].

Previous studies have found that the *CBF* genes in *Arabidopsis* and *Brachypodium* distachyon are tandemly arranged on chromosome 4 and are functional [15,44]. Since the tea plant genome has not yet been assembled to the chromosome level, we cannot currently delineate whether the *CsCBFs* are also arranged in tandem on the chromosome. CsCBFs were clustered separately from AtCBFs in the phylogenetic tree (Figure 1B), consistent with previous phylogenetic results in seven plant species [46], indicating that CBF proteins are phylogenetically conserved in many plant species. This result was further supported by conservative motif analysis, in particular, the discovery that motif 7 and motif 8 were distinctive in the CsCBF family (Figure 1C,D), which may confer unique functions to CsCBFs and should be further investigated. AtCBF4 is a unique member of the AtCBF family that regulates drought adaptation in *Arabidopsis* [19]. Intriguingly, CsCBF3 and AtCBF4 were distinguished from other homologs by clustering into different subclades, and the conserved motifs of CsCBF3 were different from those of the other four CsCBF proteins. These results suggest that CsCBF3 may be functionally unique in the CsCBF family.

Increased evidence indicates that the *CBF* genes play an important role in plant growth and development. For instance, the overexpression of *AtCBFs* leads to plant growth retardation and delayed flowering [18,29,47]. It has been proposed that CBFs inhibit plant growth by negatively regulating gibberellin synthesis, which is marked by the accumulation of DELLA proteins [48]. A recent study on *Arabidopsis cbf* triple CRISPR mutants revealed that the *CBF* genes play an important role in seedling development [5]. Tissue RNA-Seq data released with the tea plant genome will help to explore the potential function of the *CsCBFs* in plant development [36]. Our results indicate that among eight tea plant tissues, the *CsCBF* genes were mainly expressed in the winter mature leaves, roots and fruits (Figure 3). The expression data obtained from the TAIR database (https://www.arabidopsis.org/) showed that *AtCBF3* was expressed at relatively high levels in mature leaves, while the other three AtCBFs accumulated mainly in roots. In addition, light is one of the most important environmental stimuli that regulates plant development, and phytochrome-interacting factors (PIFs) play a central role in phytochrome-mediated light signaling networks [49,50,51]. Part of the PIFs can bind to the G-box and E-box cis-elements in the *AtCBF* promoter to regulate transcription [52,53,54]. Here, we identified 12 types of light-sensitive cis-elements in the *CsCBF* promoters, and the G-box elements accounted for the largest part (Figure 2). Overall, the involvement of *CsCBFs* in tea plant growth deserves further study.

All the *CsCBF* genes except *CsCBF3* were significantly induced after treatment at 4 °C in our research (Figure 4), and these results are consistent with those of previous studies [26,27]. Among the four significantly upregulated CsCBFs, there was variation in the time (from 1 h to 12 h) to reach the peak expression level under low temperature. However, three *AtCBF* genes showed constitutively high expression after transfer to low temperature and peaked within 1 h to 2 h [15]. A recent work proposed that the expression of *CBF* genes is interrelated and that some early expressed CBFs may regulate the expression of other orthologs [45]. The expression level of *CsCBF3* was too low to be reliably detected, indicating that it may be similar to *AtCBF4* that is not induced by cold [19].

The cluster analysis of putative target genes of *CsCBFs* suggests that many can be divided into 11 functional categories (Figure 6), some of which have been widely reported in previous studies [5,28,30], including a large number of target genes involved in carbohydrate metabolism, lipid metabolism, and cell wall modification. The expression levels of the carbohydrate-related genes and the accumulation of carbohydrates enhance the cold tolerance of tea plants in the winter [32]. These results are also supported by metabolomics studies in *Arabidopsis* that cold treatment or the overexpression of *AtCBF3* promotes the accumulation of glucose, raffinose, sucrose, and proline [10,47]. It has been reported that genes involved in the degradation of cell wall components may contribute to plant survival at low temperature [5]. In addition, five target genes associated with the calcium signaling pathway were identified. Cold shock has been reported to increase the level of second messenger calcium in the cytoplasm [55]. A transcriptome study revealed that calcium signaling plays an important role in tea plant responses to low temperature [31]. It is commonly known that the expression of CBF genes is regulated by the circadian clock, especially Circadian clock-associated 1 (CCA1), which binds to EE and CBS elements in the CBF promoter and contributes to cold tolerance [56,57]. Interestingly, we observed that seven putative target genes regulated by *CsCBFs* are involved in the circadian rhythm, including two circadian clock-Dof transcription factors [58,59]. Furthermore, 44 transcription factors are also regulated by *CsCBFs* (Figure 7), suggesting that the low temperature network of tea plants is subject to complex transcriptional cascade regulation. By controlling complex cascades, hormones can modulate plant response to low temperature [60]. Numerous studies have demonstrated that the expression of CBFs is regulated by GA, JA, ABA, ETH, and brassinosteroids (BRs) [60,61]. Here, a large number of plant hormone-responsive *cis*-elements were detected in the *CsCBF* promoters (Figure 2), and eleven putative target genes were involved in the plant auxin, GA, ABA, ETH, and JA hormone signaling pathways (Figure 8), which will facilitate further elucidation of the molecular regulatory mechanisms of hormones and cold signaling. Taken together, these results indicate that *CsCBFs* play functional roles by regulating target genes in multiple pathways under low temperature.

## 4. Materials and Methods

### 4.1. Plant Materials and Low-Temperature Treatment

Two-year-old potted tea plants (cv. *Tieguanyin*) grown in the Fujian Agriculture and Forestry University tea plant germplasm collection garden (Fuzhou, China) were used as the material. All tea plants used in our experiments were watered and fertilized under the same conditions and were free of pests and diseases. For the low-temperature treatment, the healthy tea plants were placed in a controlled chamber (23 °C) with a 16/8 h (day/night) photoperiod. The tea plants were low-temperature treated at 4 °C, and the second leaves were harvested at 0, 1, 3, 6, 12, 24, and 48 h after the treatment. The treatment at each time point was performed with three biological replicates, and the samples were rapidly frozen in liquid nitrogen and stored at −80 °C for subsequent analysis.

### 4.2. Identification, Sequence Alignment and Phylogenetic Analysis of CBF Homologs in Tea Plants

To obtain *CBF* gene sequences in the tea plant genome, previously published tea plant (*Camellia sinensis* var. *sinensis*) draft genome sequences (http://pcsb.ahau.edu.cn:8080/CSS/) [36] (currently available at: http://tpia.teaplant.org/index.html) were downloaded and prepared as a background file for the NCBI local BLASTn program. The coding sequences of the CBFs from *Arabidopsis* were downloaded from the Arabidopsis Information Resource (TAIR) database (https://www.arabidopsis.org/) and subjected to a BLASTn search. Furthermore, the SMART (http://smart.embl-heidelberg.de/) and CCD (https://www.ncbi.nlm.nih) online programs were used to verify the existence of the complete AP2 domain and two signature sequences for the candidate CBF protein sequence. The physical and chemical parameters of the CsCBF proteins were analyzed using the ProtParam tool from the ExPASy website (http://web.expasy.org/protparam/). Multiple sequence alignment of CBF protein sequences and their logos were analyzed by DNAman 7.0 software and WebLogo online tool (http://weblogo.berkeley.edu/logo.cgi) [62], respectively. The neighbor-joining (NJ) tree was constructed using MEGA 5.0 software (https://www.megasoftware.net/index.php) with bootstrap 1000 [63].

### 4.3. Conserved Motif Distributions and Promoter Analyses

The CBF amino acid sequences from tea plants and *Arabidopsis* were submitted to MEME Suite 5.03 (http://meme-suite.org/tools/meme) [64] to identify and visualize the conserved motifs, with the maximum number of motifs set at eight. The upstream 1000 bp *CsCBF* promoter sequences of the start codon were extracted and analyzed using TB tools version 0.665 [65] and the PlantCARE referential database (http://bioinformatics.psb.ugent.be/webtools/plantcare/html/) [66], respectively.

### 4.4. Quantitative Real-Time PCR (qRT-PCR) Analysis

Total RNA was extracted from the samples using the RNAprep pure plant kit DP441 (TIANGEN, Beijing, China), and cDNA was synthesized for qRT-PCR by the EasyScript One-Step gDNA Removal and cDNA Synthesis SuperMix Kit AE311-02 (TransGen Biotech, Beijing, China) according to the manufacturer’s protocol. qRT-PCR reactions were performed by the Trans Start^®^ Tip Green qPCR SuperMix kit AQ141-02 (TransGen Biotech, Beijing, China) in a CFX96 Touch™ Real-Time PCR detection system (Bio-Rad, Hercules, CA, USA). *CsGAPDH* (accession no. GE651107) was used as an internal control. The PCR conditions were 95 °C for 30 s, followed by 40 cycles of 95 °C for 5 s and 60 °C for 30 s. Relative gene expression levels were calculated using the 2^−ΔΔC*T*^ method [67], and the standard errors of the mean values in the replicates were calculated. The primers designed for qRT-PCR are listed in Table S6.

### 4.5. Tissue-Specific Expression Detected by RNA-seq Data

To explore the role of *CsCBF* family genes in tea plant growth and development, raw transcriptome data from eight tissues, including the root (SRX4343634), stem (SRX4343635), bud (SRX4343639), young leaf (SRX4343640), mature leaf in summer (SRX4343637), mature leaf in winter (SRX4343638), flower (SRX4343636), and fruit (SRX4343633), were downloaded from the SRA database that was previously published by Wei et al. [36]. All clean reads were mapped to the tea plant genome (*Camellia sinensis* var. *sinensis*) by TopHat2 version 2.08 [68] and then the fragments per kilobase million (FPKM) values were calculated using HTSeq version 0.9.1 [69]. Subsequently, the FPKM values of the *CsCBF* genes were clustered and visualized using TBtools version 0.665 [65].

### 4.6. Identification, Annotation, and Classification of Potential CBF Target Genes

CBF transcripts can accumulate at low temperature and regulate downstream *COR* genes by binding to the CRT/DRE motif (G/ACCGAC) in their promoters. To investigate the possible *CsCBF* target *COR* genes, previously calculated expression data for mature tea plant leaves harvested in the summer and winter were used for further analysis. Two criteria were used to identify the *CBF* target genes: (1) the genes were up- or downregulated at least 2-fold in winter mature leaves compared to summer mature leaves. (2) The genes have at least one CRT/DRE motif. TB tools version 0.665 [65] was used to extract the upstream 1000 bp gene promoter sequences of the start codon, and Analysis of Motif Enrichment (AME) tool (http://meme-suite.org/tools/ame) from MEME Suite Version 5.0.3 was used to test for the presence of the CRT/DRE motif in these genes [70]. Moreover, the CBF target gene functions were annotated using the nonredundant protein sequences (Nr) database, the Gene Ontology (GO) database, and the Kyoto Encyclopedia of Genes and Genomes (KEGG) database, and the data were analyzed and visualized on the OmicShare online platform (http://www.omicshare.com/tools). Subsequently, we manually classified and analyzed the *CBF* target genes based on their biological functions.

## 5. Conclusions

In the present study, we identified five *CsCBF* genes in the tea plant genome and analyzed their phylogenetic tree, conserved domains and motifs, and promoter composition. These results suggest that *CsCBF3* may be functionally unique in the *CsCBF* family. This is further supported by our findings that the low-temperature treatment resulted in the significant induction of all *CsCBF* genes except *CsCBF3* after treatment at 4 °C. Furthermore, we also found that *CsCBFs* play a functional role by regulating target genes in multiple pathways at low temperature, including carbohydrate metabolism, lipid metabolism, cell wall modification, hormone signaling, and calcium signaling pathways. Our findings will provide the basis for the further resolution of the functional roles of the *CsCBF* genes in tea plants.

## Figures and Tables

**Table 1:** Characterization of *CsCBFs* in tea plants.

Gene Name	Locus ID	CDS Length	Animo Acids	MW (kDa)	pI	Instability Index	Aliphatic Index	GRAVY	Subcellular Localization
*CsCBF1*	TEA010420.1	720	239	26.44	5.22	49.45	73.51	−0.28	nucleus
*CsCBF2*	TEA010423.1	540	179	19.54	9.54	46.70	74.75	−0.40	nucleus
*CsCBF3*	TEA010806.1	753	250	27.63	6.10	54.32	66.40	−0.59	nucleus
*CsCBF4*	TEA011105.1	777	258	27.79	4.94	49.28	65.12	−0.47	nucleus
*CsCBF5*	TEA031249.1	1056	351	38.28	4.75	47.30	66.55	−0.48	nucleus

## Supplementary Materials

Supplementary materials can be found at https://www.mdpi.com/1422-0067/20/20/5137/s1.

## Abbreviations

CBFs	C-repeat binding factors
CRT/DRE	C-repeat/dehydration-responsive
COR	Cold-responsive gene
AP2/ERF	APETALA2/Ethylene-Responsive Factor
FPKM	Fragments Per Kilobase of transcript per Million mapped reads
AME	Analysis of Motif Enrichment
Nr	Nonredundant protein sequences
GO	Gene Ontology
KEGG	Kyoto Encyclopedia of Genes and Genomes
CDS	Coding sequences
GA	Gibberellin
ABA	Abscisic acid
ETH	Ethylene
JA	Jasmonic acid
