# Supplementary figures and images for: Identification of CBF Transcription Factors in Tea Plants and a Survey of Potential CBF Target Genes under Low Temperature
Source: Int J Mol Sci. 2019 Oct 17;20(20):5137. doi: 10.3390/ijms20205137 (PMC6829267; doi:10.3390/ijms20205137)

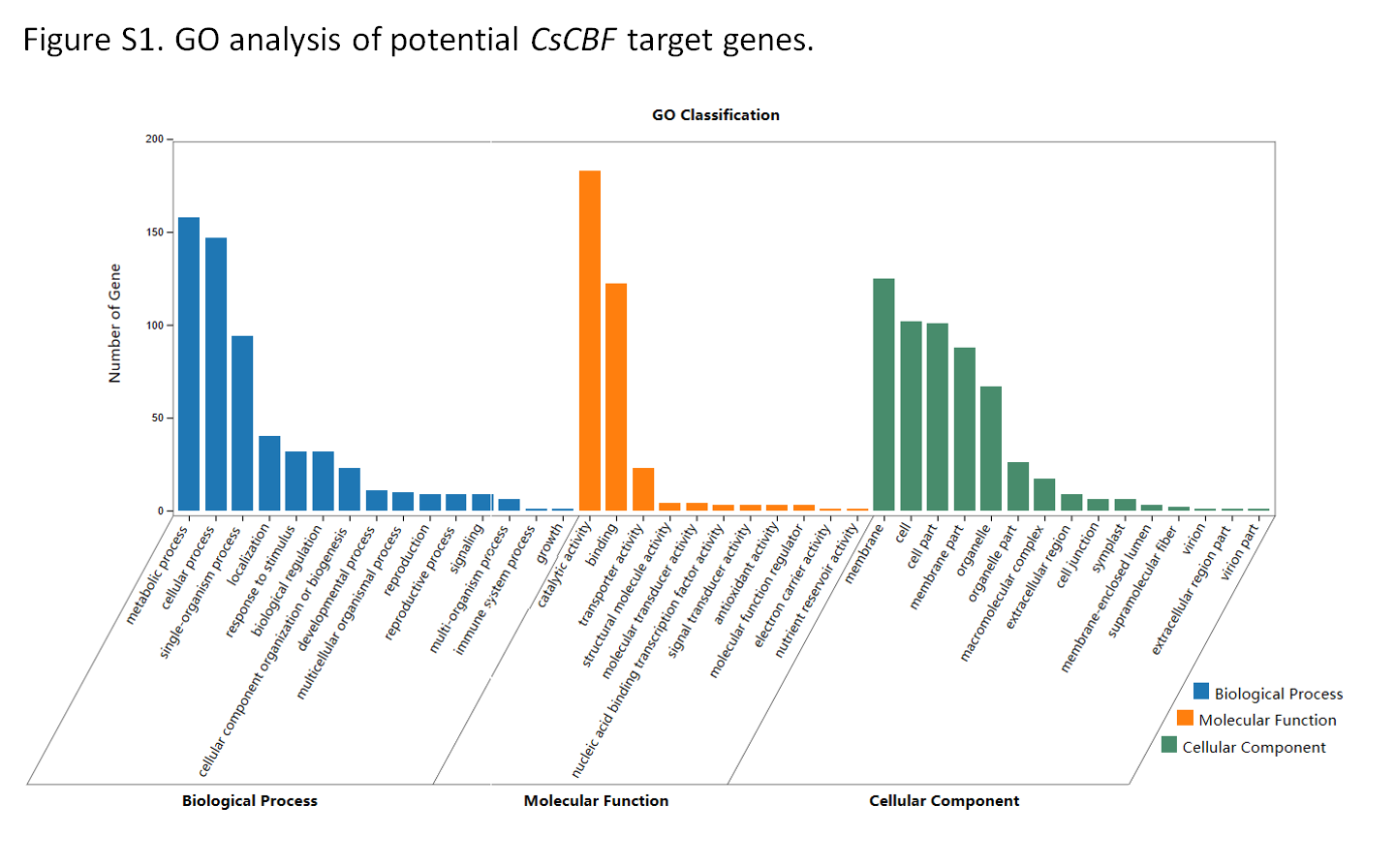

Supplement: Supplementary file 1 [file ijms-20-05137-s001.zip › Supplementary Materials/Figure S1.tif]

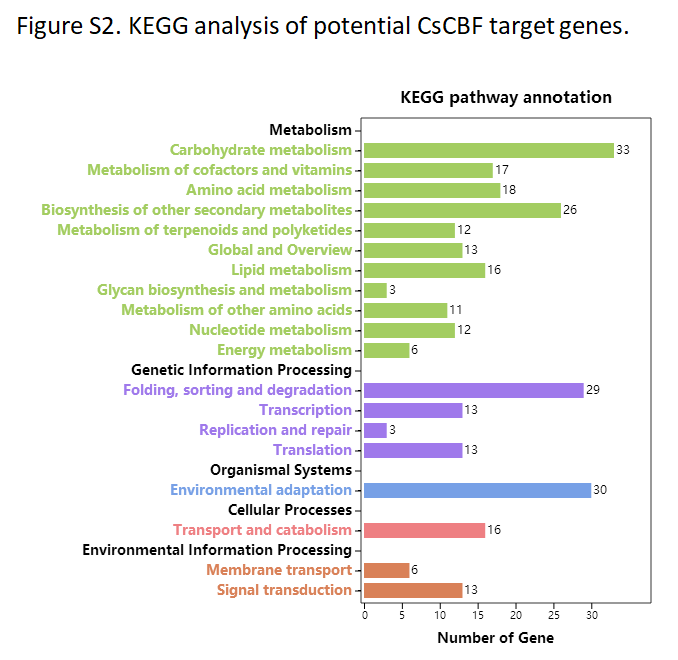

Supplement: Supplementary file 1 [file ijms-20-05137-s001.zip › Supplementary Materials/Figure S2.tif]
